# Genotypic and Environmental Variations in Kernel Color Indices in the Main Almond (*Prunus dulcis* (Mill.) D.A. Webb) Cultivars Grown in North-Eastern Morocco

**DOI:** 10.1155/2021/9970223

**Published:** 2021-05-10

**Authors:** El Hassan Sakar, Mohamed El Yamani, Abdelali Boussakouran, Yahia Rharrabti

**Affiliations:** ^1^Department of Biology, Faculty of Sciences of Tetuan, Abdelmalek Essaâdi University, Mhannech II. 93002, Tetuan, Morocco; ^2^Laboratory of Natural Resources and Environment, Polydisciplinary Faculty of Taza, Sidi Mohamed Ben Abdellah University, B. P 1223, Taza, Morocco; ^3^Essaouira School of Technology, Cadi Ayyad University, Km 9, Route d'Agadir, BP. 383, Essaouira Al Jadida, Morocco

## Abstract

Color indices are important quality traits that define the consumer visual acceptance and agroindustrial preferences. Little is known regarding these properties in the commercial almond cultivars grown in Morocco. This work aimed at investigating kernel color indices in five cultivars, namely, “Fournat de Brézenaud,” “Tuono,” “Ferragnès,” “Ferraduel,” and “Marcona.” Color indices consisted in the following: brightness (*L*^∗^), redness index (*a*^∗^), yellowness index (*b*^∗^), chroma (*C*^∗^), hue (*H*^∗^), and metric saturation (*S*^∗^). Measurements were performed over three consecutive growing seasons (2016–2018) across five different sites from northern (Aknoul, Bni Hadifa, and Tahar Souk) and eastern (Rislane and Sidi Bouhria) Morocco. All factors (cultivar, growing season, and site) affected significantly studied color properties; however, genotype was the main variability source. Wide variabilities were found among cultivars. “Marcona” showed the highest *L*^∗^, while “Ferragnès” and “Ferraduel” displayed greater scores of *a*^∗^, *b*^∗^, *C*^∗^, and *S*^∗^. Sidi Bouhria presented the lowest *L*^∗^ but higher *a*^∗^, *H*^∗^, and *S*^∗^. Moreover, Bni Hadifa displayed higher *L*^∗^, *b*^∗^, and *C*^∗^. 2016 (drier growing season) had the highest values of most indices. Principal component analyses (PCA) discriminate all factors through the first three components: PC1 (61%, genetic component) and PC2 (30%) and PC3 (7%) which were of environmental nature since they separate sites and growing seasons, respectively. Despite environmental effects, we suggested a possible discrimination among the studied cultivars based on their kernel color indices. Drought conditions during fruit development seemed to improve kernel quality via synthesis of pigments resulting in higher *a*^∗^ and *b*^∗^.

## 1. Introduction

Cultivated almond (*Prunus dulcis* (Miller) D.A. Webb) is one of the most important nut crops worldwide from both an agronomic and economic point of view. According to the last statistic releases of FAOSTAT [1], the global almond in-shell production reached 2239697 tons coming from an area harvested of 1925887 hectares. The value of global production is estimated to be around 13524.44 million US$.

Nut consumption (including almonds) is highly linked to numerous health benefits including maintenance of healthy blood lipid levels, reduction of the heart disease risk, and lower incidence of metabolic syndrome [2–4]. All these benefits are due to the richness of almonds in several valuable nutrients including fatty acids, proteins, minerals, dietary fibers, and vitamins, among others [5–9].

A literature review shows that a huge number of research studies were devoted to assess the almond quality for both nut and kernel in northern and southern hemispheres [8, 10]. Along with chemical composition of almond kernel, quality is also defined by the physical traits. The physical fruit traits must be included to characterize almond fruit [11, 12], since these traits not only serve for classification purposes but also affect industrial processing and consumer acceptance. As reported by several other authors, physical quality traits encompass geometrical, gravimetrical, frictional, and tegument color [11, 13–16]. These traits depend upon several factors such as geographic origin, genotype, agronomical managements under which almonds are grown, and moisture level [11, 13].

In almond fruit, the tegument known also as pellicle, almond skin, or seed coat represents about 4% of an almond fruit. It protects the almond kernel from microbial contamination and oxidation [17]. Several food applications of almonds in bakery, confectionary items, snack formulations, cereals, and marzipan require the almond kernel alone without the tegument [17]. However, the almond tegument is a valuable agricultural by-product due to its richness in phenolic compounds as reported by several studies [17–19]. Owing to its higher antioxidant power, the almond tegument is widely reported to be a good source of phenolic compounds for various uses [18–20].

Tegument color is reported to be important as one of the physical fruit traits [12, 21–23]. It is generally assessed using the CIELAB color space (known also as CIE *L*^∗^*a*^∗^*b*^∗^). In this space, color is defined as three numerical values known as the trichromatic coordinates (*L*^∗^, *a*^∗^, and *b*^∗^). The coordinate *L*^∗^ refers to the lightness of a given sample (scored from 0 which represents the black color to 100 representing the white one); *a*^∗^ is the coordinate that defines the degree of approximation to the red color when *a*^∗^ takes positive values and green when negative; and the coordinate *b*^∗^ indicates yellow when it takes positive values and blue when negative^23^. Furthermore, chroma (*C*^∗^) and hue angle (*H*^∗^) are other color indices calculated on the basis of *L*^∗^, *a*^∗^, and *b*^∗^. Following the work of McGuire [24], *C*^∗^ is a measurement of chromaticity, which defines the purity or saturation of the color and *H*^∗^ describes the color nuance as follows: red-purple: 0°, yellow: 90°, bluish-green: 180°, and blue: 270°.

Although color is one of the most important attributes used by consumers to distinguish a product quality [25], USDA standards for grading almond kernels do not describe kernel color as a means for the distinction of varietal character [26]. In contrast, consumer preferences have an important role in determining marketability of a given agricultural product [27], and almond kernels of dark color can be perceived as having lost freshness or turned rancid. This reaction is delayed or even reduced by the presence, in the almond pellicle [17–19], of antioxidative phenolic compounds (such as *α*-tocopherol) which act as free-radical scavengers.

Besides, freshly shelled almond kernels display varietal differences in tegument color [22, 23], which are genetically controlled [28] with a heritability value of 0.42 as outlined in the work of Gradziel and Martínez-Gómez [29]. Environmental conditions under which almonds are grown were reported to affect kernel color. In this context, a study involving the major Californian almond cultivar “Nonpareil” during seven harvest seasons indicated that all three coordinates related to tegument color varied significantly across harvest seasons [30]. Moreover, Valverde et al. [11] reported that values of coordinates and attributes of the CIELAB color system vary as functions of the climatic conditions in each harvest season, but not as a function of fertilizer treatment or irrigation regime. All these findings give evidence about the genotypic and environmental effects on tegument color coordinates.

Given the importance of Nonpareil Marketing Group Cultivars owing to its higher commercial value and consumer appreciation, in a study involving 6 Californian cultivars during 2 harvest seasons, Ledbetter and Sisterson [22] used some kernel physical traits to investigate the possibility of distinguishing Nonpareil Marketing Group Cultivars (“Nonpareil,” “Jeffries,” “Kapareil,' and “Milow”) from cvs “Carmel” and “Padre” representative of the California Marketing Group and the Mission Marketing Group, respectively. Following these authors, kernel brightness is the most discriminating trait, regardless of the character set used in analyses.

Besides, after harvest, the almond tegument is subjected to darkening during storage for long term and, therefore, affects the stored almonds marketability. The darkening extent during the period of storage is controlled by both storage conditions and the genetic component [21]. These authors observed a degradation of almond tegument color coordinates under various storage temperatures with differential responses among almond accessions involved in this study. Likewise, the degree of tegument color degradation varied as a function of the storage temperature. Nizamlioglu and Nas [31] investigated kinetic of color changes during roasting and storage in the Turkish variety “Akbadem,” and they found that *L*^∗^ and *H*^∗^ values tended to decrease linearly during roasting. A similar trend was observed for *L*^∗^, *C*^∗^, *a*^∗^, *b*^∗^, and *S* (metric saturation) during storage.

From a pomological standpoint, almond quality definition must take into account both compositional and physical fruit traits, and limited research works have been conducted to characterize the physical fruit traits in commercial almond cultivars grown in Morocco, especially the effect of genotype × environment interaction on tegument color variation, hence the originality of this paper. Therefore, the objectives of this work were to (i) evaluate color indices in almond kernels of the main cultivars grown commercially in north-eastern Morocco (ii) and to assess to what extent unpredictable weather conditions and edaphic factors during nut development affect kernel color in these almond cultivars.

## 2. Materials and Methods

### 2.1. Study Sites

This study was conducted across five sites, which are located in the main almond production regions of north-eastern Morocco. Sites were chosen to cover a range of different environments (altitudinal and climatic). In the five sites, orchards were conducted under rainfed conditions and underwent similar agronomical practices.

Central northern Morocco was represented by the following sites: Aknoul (34°39′0″ N, 3°52′0″ W; 955 m.a.s.l.), Bni Hadifa (35°1′22″ N, 4°8′27″ W; 891 m.a.s.l.), and Tahar Souk (35°1′22″ N, 4°8′27″ W; 994 m.a.s.l.). Eastern Morocco was represented by two sites: Rislane (34 46′ 23″ N, 2 27′ 44″ W; 690 m.a.s.l.) and Sidi Bouhria (34° 44′ 21″ N 2° 21′ 44″ W; 623 m.a.s.l.).

### 2.2. Plant Material and Sampling

Plant material consisted in five almond commercial cultivars (“Ferraduel,” “Ferragnès,” “Fournat de Brézenaud,” “Marcona,” and “Tuono”), which are widely grown in all sites described above.

Three trees from each cultivar were marked and used as replicates in the five studied sites. Sampling was carried out at the physiological maturity stage, which fits 89 on the BBCH (Biologische Bundesanstalt, Bundessortenamt und Chemische Industrie) phenological scale as described by Meier [32] and adapted by Sakar et al. [33]. We harvested about 1.5 kg of fruits around the canopy from each of all marked trees across the five sites during the three consecutive growing seasons (2016−2018). Samples were immediately brought to laboratory using black polyethylene bags. Almonds were cracked with a hammer and manually shelled in controlled conditions for immediate drying [34].

### 2.3. Kernel Color Determinations

From each sample described above, a subsample of 30 almonds was selected to determine the almond kernels' color. After that, almond nuts were cracked using a hammer to release almond kernels. Immediately after cracking, tegument color indices were analyzed by measuring reflected color in the CIELAB (*L*^∗^, *a*^∗^, *b*^∗^) color system according to the work of Valverde et al. [11] using a handheld tristimulus colorimeter (chroma meter model CR-400, Konica Minolta manufacturer, Tokyo, Japan).

Tegument color indices measured consisted in the following: the brightness (*L*^∗^) which varies between 0 (black) and 100 (white) and the coordinates of opposed color *a*^∗^ and *b*^∗^ whose variation are between −60 and +60. The redness (*a*^∗^) assigns positive values for the red and negative values for green. With respect to yellowness (*b*^∗^), positive values are assigned to yellow and negative ones to blue [35].

Chroma (*C*^∗^) and hue angle (*H*∗) were computed according to the equations given by McGuire [24]:

(1) $$\begin{array}{c} C^{*}=\sqrt{{\left(a^{*}\right)}^{2}+{\left(b^{*}\right)}^{2}},\\ H*=\text{acrtan }g\left(\frac{b^{*}}{a^{*}}\right). \end{array}$$

Metric saturation (*S*∗) was calculated following the equation given by Valverde et al. [11]:

(2) $$\begin{array}{c} S^{*}=\frac{{\left(a^{*}\right)}^{2}+{\left(b^{*}\right)}^{2}}{L^{*}}, \end{array}$$

where *L*^∗^: brightness index, *a*^∗^: redness index, and *b*^∗^: yellowness index.

### 2.4. Statistical Analysis

All measurements and calculations were carried out in triplicate. The data obtained were subjected to statistical analysis by means of STATGRAPHICS package version XVIII (Statpoint Technologies, Inc., Virginia, USA). Analyses of variance were computed using the general linear model procedure. Mean comparisons between sites, cultivars, and growing seasons were carried out using the Least Significant Difference (LSD) test at 5% as the probability level. Principal component analysis (PCA) was performed on mean values to discriminate among sites, cultivars, and growing seasons. A correlation matrix was also calculated using mean values.

## 3. Results

### 3.1. Analyses of Variance

The outcomes of the combined analysis of variance for the whole investigated kernel color indices are given in Table 1. From these outcomes, all factors (cultivar, site, and growing season) and mainly site × interaction affected significantly all investigated parameters. In addition, cultivar was the main variability source for brightness index (*L*^∗^), redness index (*a*^∗^), yellowness index (*b*^∗^), and chroma (*C*^∗^) since it explained around 59% of their variance. Hue angle (*H*^∗^) was mainly under site effect which allowed to explain 61% of its total variability. Growing season effect explained about 27% of total variance in redness index (*a*^∗^) and 14% for the remaining traits. In addition, around 22% of the total variance in the brightness (*L*^∗^) and yellowness (*b*^∗^) indices was attributed to site effect. Regarding interactions, only site by cultivar was important since it explained about 7% of the total variance in our data. The remaining interactions were of lower extent, and they explained together less than 1% of the total variance.

### 3.2. Genotypic Effects on Kernel Color Indices

Table 2 shows mean values of cultivars for brightness index (*L*^∗^), redness index (*a*^∗^), yellowness index (*b*^∗^), chroma (*C*^∗^), hue angle (*H*^∗^), and metric saturation (*S*^∗^) in the five studied almond cultivars. As it can be seen in this table, significant variations were highlighted among these cultivars. Moreover, “Marcona” showed the greatest value of brightness index (*L*^∗^ = 48.41), and the lowest one was displayed by “Tuono” (42.13). “Ferraduel” was distinguished by the highest scores of redness index (*a*^∗^ = 19.29), yellowness index (*b*^∗^ = 31.25), chroma (*C*^∗^ = 36.73), and metric maturation (*S*^∗^ = 28.11). The greatest value of hue angle (*H*^∗^ = 55.92) was found in “Tuono,” which was also found to have the lowest score of yellowness index (*b*^∗^ = 28.14). The smallest records of redness index (*b*^∗^ = 17.04), hue angle (*H*^∗^ = 53.15), and metric saturation (*S*^∗^ = 23.38) were presented by “Marcona.” “Ferragnès” and “Fournat de Brézenaud” were found to display medium values for almost color indices between the remaining cultivars.

### 3.3. Site Effects on Almond Kernel Color Indices

Mean values of sites for brightness index (*L*^∗^), redness index (*a*^∗^), yellowness index (*b*^∗^), chroma (*C*^∗^), hue angle (*H*^∗^), and metric saturation (*S*^∗^) of in the investigated almond cultivars are summarized in Table 2. Between the studied sites, there were wide variabilities for the majority of color indices. Northern sites were found to have higher values of brightness index (*L*^∗^), yellowness index (*b*^∗^), chroma (*C*^∗^), and metric saturation (*S*^∗^). Values of redness index (*b*^∗^) and hue angle (*H*^∗^) were higher in sites of eastern Morocco. The highest scores of brightness index (*L*^∗^ = 47.39), yellowness index (*b*^∗^ = 30.27), and chroma (*C*^∗^ = 35.16) were found in Bni Hadifa (northern Morocco). In contrast, Sidi Bouhria was marked by the greatest record of redness index (18.46) and hue angle (*H*^∗^ = 56.79). The lowest record of yellowness index (*b*^∗^ = 28.14), chroma (*C*^∗^ = 31.31), and metric saturation (*S*^∗^ = 24.83) were displayed by Rislane. Sidi Bouhria was found to have the smallest value of brightness index (*L*^∗^ = 44.48). Aknoul had the lowest value of redness index (*a*^∗^ = 17.61) and hue angle (*H* = 52.88).

### 3.4. Growing Season Effects on Kernel Color Indices

Mean values of growing seasons for brightness index (*L*^∗^), redness index (*a*^∗^), yellowness index (*b*^∗^), chroma (*C*^∗^), hue angle (*H*^∗^), and metric saturation of the investigated almond cultivars are shown in Table 2. There were significant variations among the three growing seasons for almost all color indices. Furthermore, the 2016 growing season was marked by the highest score of all color indices: brightness index (*L*^∗^ = 46.64), redness index (*a*^∗^ = 18.53), yellowness index (*b*^∗^ = 29.87), chroma (*C*^∗^ = 35.16), hue angle (*H*^∗^ = 55.53), and metric saturation (*S*^∗^ = 26.57). In contrast, the 2018 growing season was found to display the smallest value of brightness index (*L*^∗^ = 44.87), redness index (*a*^∗^ = 17.51), yellowness index (*b*^∗^ = 28.89), chroma (*C*^∗^ = 33.81), hue angle (*H*^∗^ = 54.49), and metric saturation (*S*^∗^ = 25.16).

### 3.5. Correlations among Studied Traits

The correlation matrix among the studied parameters is shown in Table 3. As evidenced in this table, important correlations were highlighted between almost color indices. Redness index was positively correlated with yellowness index (*r* = 0.751^∗∗^). Chroma (*C*^∗^) was positively linked to redness index (*r* = 0.873^∗∗∗^) and yellowness index (*r* = 0.978^∗∗∗^). Metric saturation was associated negatively with brightness index (*r* = −0.308^∗^) and positively with redness index (*r* = 0.823^∗∗∗^), yellowness index (*r* = 0.666^∗∗^), chroma (*r* = 0.754^∗∗^), and hue angle (*r* = 0.362). Redness index and hue angle were positively linked to each other (*r* = 0.511^∗^). The remaining correlations were of lesser extent and insignificant.

### 3.6. Principal Component Analysis (PCA)

**Figure 1:**
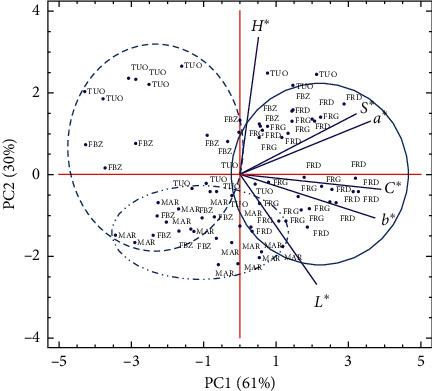
Principal component analysis (PCA) projections on PC1 and PC2. The eigenvalues are symbolized as blue segments representing traits that most affect each principal component. The 75 points plotted are cultivar mean values of each studied kernel color index: brightness index (*L*^∗^), redness index (*a*^∗^), yellowness index (*b*^∗^), chroma (*C*^∗^), hue angle (*H*^∗^), and metric saturation (*S*^∗^) of five almond cultivars grown in different sites of north-eastern Morocco (aknoul, bni hadifa, tahar souk, rislane, and sidi bouhria) during three growing seasons (2016−2018). FRD = “Ferraduel,” FRG = “Ferragnès,” FBZ = “Fournat de Brézenaud,” MAR = “Marcona,” and TUO = “Tuono.”

PCA was used as a multivariate method to better discriminate between cultivars, sites, and growing seasons. The three first principal components (PCs) were retained because they allowed explaining 98% of the total variability. PC1, PC2, and PC3 accounted for 61%, 30%, and 7%, respectively. Points plotted on the surface delimited by axis 1 and 2 (Figure 1) are related to cultivars, which seem to be distributed along PC1 (genetic component). As evidenced in this figure, “Ferragnès” and “Ferraduel” plotted on the positive direction of PC1 were associated with great values of redness index (*a*^∗^), yellowness index (*b*^∗^), chroma (*C*^∗^), and metric saturation (*S*^∗^). Most points corresponding to the set of cultivars “Fournat de Brézenaud,” “Tuono,” and “Marcona” were plotted on the negative side of PC1. Furthermore, “Tuono” and “Marcona” interacted with the highest score of hue angle (*H*^∗^) and brightness index (*L*^∗^), respectively.

**Figure 2:**
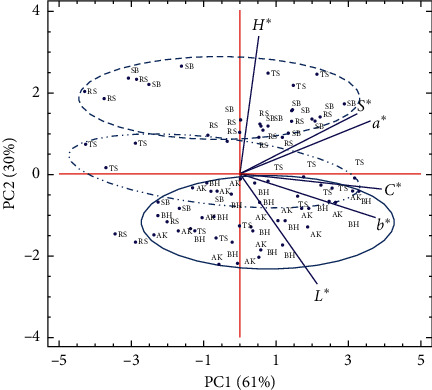
Principal component analysis (PCA) projections on PC1 and PC2. The eigenvalues are symbolized as blue segments representing traits that most affect each principal component. The 75 points plotted are cultivar mean values of each studied kernel color index: brightness index (*L*^∗^), redness index (*a*^∗^), yellowness index (*b*^∗^), chroma (*C*^∗^), hue angle (*H*^∗^), and metric saturation (*S*^∗^) of five almond cultivars grown in different sites of north-eastern Morocco (Aknoul, Bni Hadifa, Tahar Souk, Rislane, and Sidi Bouhria) during three growing seasons (2016−2018). AK = Aknoul, BH = Bni Hadifa, TS = Tahar Souk, RS = Rislane, and SB = Sidi Bouhria.

Similarly, points plotted on the plan determined by axis 1 and 2 were related to sites (Figure 2). PC2 appears to discriminate between sites of northern Morocco (Aknoul, Bni Hadifa, and Tahar Souk) towards the negative direction and eastern sites (Rislane and Sidi Bouhria) towards the positive side of this second axis (environmental component). Northern sites exhibited higher scores of yellowness index (*b*^∗^), brightness index (*L*^∗^), and chroma (*C*^∗^). In contrast, eastern sites interacted with higher values of redness index (*a*^∗^), hue angle (*H*^∗^), and metric saturation (*S*^∗^).

**Figure 3:**
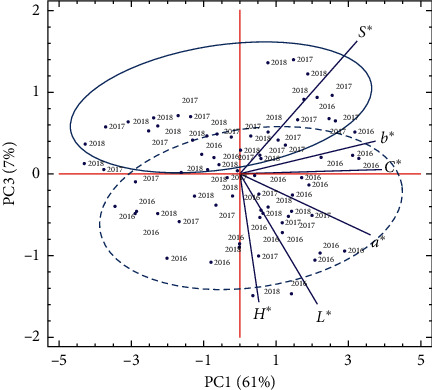
Principal component analysis (PCA) projections on PC1 and PC3. The eigenvalues are symbolized as blue segments representing traits that most affect each principal component. The 75 points plotted are cultivar mean values of each studied kernel color index: brightness index (*L*^∗^), redness index (*a*^∗^), yellowness index (*b*^∗^), chroma (*C*^∗^), hue angle (*H*^∗^), and metric saturation (*S*^∗^) of five almond cultivars grown in different sites of north-eastern Morocco (Aknoul, Bni Hadifa, Tahar Souk, Rislane, and Sidi Bouhria) during three growing seasons (2016−2018). *2016* = 2016 growing season, *2017* = 2017 growing season, and *2018* = 2018 growing season.

Figure 3 presents the distribution of growing seasons on the surface determined by PC1 and PC3. The third component, which accounted for about 7% of the total variability, separated between the three growing seasons (environmental component). In fact, most of points corresponding to the 2016 growing season were plotted towards the negative side of PC3 and interacted with the higher scores of kernel color indices (*L*^∗^, *a*^∗^, *b*^∗^, *C*^∗^, and *H*^∗^), while 2017 and 2018 growing seasons were distributed on the positive side of this component and interacted with higher score of metric maturation (*S*^∗^) and lower and medium values of remaining indices. The outcomes highlighted here by the PCA approach (Figures 1to 3) confirmed the results of both ANOVA and mean comparisons already reported in Tables 1 and 2.

## 4. Discussion

Color indices are reported to be one of the most important quality traits that must be taken into account when assessing kernel quality [12, 21–23, 30, 36]. Here, we reported about kernel color indices in the main almond cultivars grown across a range of sites from the north and the east of Morocco over three growing seasons. On the basis of our results, all factors (cultivar, growing season, and site) and mostly site × cultivar interaction impacted significantly all kernel color indices. However, cultivar effect was the main variability source in our data. Similar trends were reported by several authors for almonds grown under various conditions in Spain [11], Iran [12], Portugal [23], and USA [21, 22]. Kernel color is quantitatively inherited [37] with a heritability value of 0.42 according to Kester et al. [38]. Following these authors, growing season effect was of lesser extent (around 17% of the total variance of kernel color) in agreement with our outcomes. Despite the environmental effects, kernel color remains a typical varietal trait with higher heritability as outlined in several reports [28, 39].

In a study conducted during two growing seasons, Ledbetter and Sisterson et al. [22] focused on distinguishing some Californian cultivars using physical carpological traits. They found that kernel luminosity (brightness, *L*^∗^) is the most discriminative trait among studied cultivars. In the case of our studied cultivars, as evidenced in the results section, the three basic color indices brightness (*L*^∗^), redness (*a*^∗^), and yellowness (*b*^∗^) were found to be significantly different suggesting the possibility of their discrimination on the basis of kernel color. Similar trends were reported for 8 commercial cultivars grown under Portuguese conditions^23^.

As outlined in the results section, we found wide variabilities, between sites, regarding kernel color indices. Valverde et al. [11] reported similar results for almond “Guara” grown under different fertilizers (organic and inorganic) and water regimes (drip-irrigation and nonirrigation). As highlighted by Yaghini et al. [12], the basic color indices are related to the kinds and quantities of pigments accumulated in fruits. Likewise, some authors found good correlations between values of redness index (*a*∗) and hue angle (*H*∗) on one hand and the carotenoid concentration on the other hand [40, 41]. Among sites, variabilities in terms of color indices could be attributed to soil fertility differences among these sites in agreement with results reported by Ames et al. [42] for peach grown under different rates of nitrogen fertilization.

As for sites, we reported also significant variations between growing seasons. These findings were in agreement with what was previously published in many works [11, 22, 30]. Following the work of Ledbetter and Sisterson [30], yearly variations in kernel color could be due to temperature variations. In our results, as compared to the two growing seasons, 2018 was marked by later ripening and harvesting. This could expose fruits to higher temperatures in late summer resulting in more dark kernels as explained by Ledbetter and Sisterson [30]. The 2016 growing season was characterized by rainfall scarcity and higher values of most studied kernel color indices. Similar trends were outlined by Valverde et al. [11], who reported higher values of these indices under nonirrigation conditions.

As highlighted in the results section, significant correlations were reported between kernel color indices. Among the three basic indices, redness index (*a*^∗^) and yellowness index (*b*∗) were positively linked. These associations were in agreement with findings of Valverde et al. [11] for “Guara.” Following these authors, this correlation remains positive under various water regimes and fertilizer treatments. As explained by Cantín et al. [43], fruit quality traits are controlled by major genes and quantitively inherited. Following Hansche et al. [44], correlations between such traits could be partially explained by the pleiotropic effects or linkages that exist among the genes encoding for these traits.

PCA is a multivariate statistical analysis used with an emphasis on the reduction of variables that most explain data variability [45–55]. Indeed, several reports used the PCA approach in order to discriminate among cultivars, growing seasons, and growing areas based on physical fruit traits [12, 22, 56, 57]. In our data, the first component, whose magnitude exceeded 60% of data variability, seemed to be of genetic nature since it separated among cultivars. Together, the second and the third component were of environmental extent (about 37% of total variability) since they discriminated among growing seasons and sites. Ledbetter and Sisterson [22] and Summo et al. [58] tried to discriminate among several commercial cultivars using PCA as a multivariate statistical analysis combined with physical fruit traits. These authors found that kernel brightness is the most discriminative physical trait for the investigated cultivars confirming the predominance of genetic control of this index.

## 5. Conclusions

Despite the genetic control in the main kernel color indices studied here, environmental conditions accounted for a large extent of variability in the obtained results. On the basis of our results, more dry areas with higher temperatures during fruit ripening resulted in kernels of lower values of brightness (darker kernels) but higher redness and yellowness indices and chroma. Results obtained for the three basic color indices (brightness, redness, and yellowness) suggest the possibility of their use to discriminate among the studied cultivars.

## Figures and Tables

**Table 1:** Mean squares of the combined analyses of variance for brightness index (*L*^∗^), redness index (*a*^∗^), yellowness index (*b*^∗^), chroma (*C*^∗^), hue angle (*H*^∗^), and metric saturation (*S*^∗^) of five almond cultivars grown in different sites of north-eastern Morocco (Aknoul, Bni Hadifa, Tahar Souk, Rislane, and Sidi Bouhria) during three growing seasons (2016−2018).

Source of variation	Df	*L* ^∗^	*a* ^∗^	*b* ^∗^	*C* ^∗^	*H* ^∗^	*S* ^∗^
Growing season (GS)	2	59.404^*∗∗*^	20.191^*∗∗∗*^	18.096^*∗∗∗*^	34.685^*∗∗∗*^	30.24^*∗∗∗*^	38.5^*∗∗∗*^
Site (*S*)	4	90.267^*∗∗*^	4.643^*∗∗*^	35.811^*∗∗∗*^	23.772^*∗∗∗*^	137.76^*∗∗∗*^	32.7^*∗∗∗*^
Cultivar (*C*)	4	292.288^*∗∗∗*^	43.353^*∗∗∗*^	81.572^*∗∗∗*^	119.394^*∗∗∗*^	50.56^*∗∗∗*^	152.42^*∗∗∗*^
Replicate (*S*)	10	0.001	0.002	0.001	0.001	0.02	3.6
GS × *S*	8	0.307^*∗*^	0.065^*∗*^	0.124^*∗*^	0.097^*∗*^	0.79^*∗*^	3.9
GS × *C*	8	0.002	0.006	0.013^*∗*^	0.009^*∗*^	0.09	3.4
*S* × *C*	16	11.978^*∗∗*^	6.432^*∗∗*^	10.982^*∗∗*^	16.607^*∗∗∗*^	6.53^*∗*^	30.4^*∗∗∗*^
GS × *S* × *C*	32	0.002	0.009	0.014^*∗*^	0.007^*∗*^	0.12	3.6
Residual	140	0.001	0.002	0.001	0.001	0.02	3.5
Total	224						

^*∗*^Significant at 0.05 probability level; ^*∗∗*^significant at 0.01 probability level; ^*∗∗∗*^significant at 0.001 probability level.

**Table 2:** Mean values of cultivars, sites, and growing seasons for brightness index (*L*^∗^), redness index (*a*^∗^), yellowness index (*b*^∗^), chroma (*C*^∗^), hue angle (*H*^∗^), and metric saturation (*S*^∗^) of five almond cultivars grown in different sites of north-eastern Morocco (Aknoul, Bni Hadifa, Tahar Souk, Rislane, and Sidi Bouhria) during three growing seasons (2016−2018). Means for each character followed by the same letter are not significantly different at *P* < 0.05.

	*L* ^∗^	*a* ^∗^	*b* ^∗^	*C* ^∗^	*H* ^∗^	*S* ^∗^
*Cultivar*						
“Ferraduel”	46.97 c	19.29 a	31.25 a	36.73 a	55.32 b	28.11 a
“Ferragnès”	47.08 b	18.69 b	30.38 b	35.68 b	55.15 c	27.04 b
“Fournat de Brézenaud”	44.16 d	17.19 d	28.35 d	33.16 e	54.46 d	24.93 d
“Marcona”	48.41 a	17.04 e	28.99 c	33.63 c	53.15 e	23.38 e
“Tuono”	42.13 e	17.61 c	28.14 e	33.19 d	55.92 a	26.23 c

*Site*						
Aknoul	47.19 b	17.61 e	30.12 b	34.89 b	52.88 e	25.83 bc
Bni Hadifa	47.39 a	17.89 c	30.27 a	35.16 a	53.36 d	25.51 cd
Tahar Souk	44.91 c	18.03 b	29.67 c	34.73 c	54.61 c	27.01 a
Rislane	44.78 d	17.81 d	28.14 e	33.31 e	56.37 b	24.83 d
Sidi Bouhria	44.48 e	18.46 a	28.91 d	34.31 d	56.79 a	26.53 ab

*Growing season*						
2016	46.64 a	18.53 a	29.87 a	35.16 a	55.53 a	26.57 a
2017	45.74 b	17.84 b	29.50 b	34.48 b	54.38 b	26.08 a
2018	44.87 c	17.51 c	28.89 c	33.81 c	54.49 b	25.16 c

**Table 3:** Coefficients of correlation among the studied traits: brightness index (*L*^∗^), redness index (*a*^∗^), yellowness index (*b*^∗^), chroma (*C*^∗^), hue angle (*H*^∗^), and metric saturation (*S*^∗^) of five almond cultivars grown in different sites of northern Morocco (Aknoul, Bni Hadifa, Tahar Souk, Rislane, and Sidi Bouhria) during three growing seasons (2016−2018). ^*∗*^, ^*∗∗*^, and ^*∗∗∗*^ indicate significance at 0.05, 0.01, and 0.001 levels of probability, respectively.

	*L* ^∗^	*a* ^∗^	*b* ^∗^	*C* ^∗^	*H* ^∗^	*S* ^∗^
*L* ^∗^		0.085	0.139	0.131	−0.051	−0.308^*∗*^
*a* ^∗^			0.751^*∗∗*^	0.873^*∗∗∗*^	0.511^*∗*^	0.823^*∗∗∗*^
*b* ^∗^				0.978^*∗∗∗*^	−0.185	0.666^*∗∗*^
*C* ^∗^					0.026	0.754^*∗∗*^
*H* ^∗^						0.362^*∗*^
*S* ^∗^						

## Data Availability

The data used to support the findings of this work are available, upon request, from the corresponding author.
